# Atypical Late-Onset Immune Dysregulation, Polyendocrinopathy, Enteropathy, X-Linked Syndrome with Intractable Diarrhea: A Case Report

**DOI:** 10.3389/fped.2017.00267

**Published:** 2017-12-12

**Authors:** Ting Ge, Yizhong Wang, Yanran Che, Yongmei Xiao, Ting Zhang

**Affiliations:** ^1^Department of Gastroenterology, Hepatology, and Nutrition, Shanghai Children’s Hospital, Shanghai Jiao Tong University, Shanghai, China

**Keywords:** immune dysregulation, polyendocrinopathy, enteropathy, X-linked, forkhead box protein 3, late-onset, diarrhea, hematopoietic stem cell transplantation

## Abstract

Immune dysregulation, polyendocrinopathy, enteropathy, X-linked (IPEX) syndrome is a rare life threatening congenital autoimmune disorder caused by mutations in the forkhead box protein 3 (FOXP3) gene. The main typical clinical manifestations of IPEX are enteropathy, type 1 diabetes mellitus, and skin diseases, which usually appear in the first months of life and cause death without treatment. Here, we report a 6-year-old boy with late-onset IPEX syndrome due to a c.1190G>A (p. R397Q) mutation in exon 11 of the FOXP3 gene. The boy had intractable diarrhea, abdominal pain, recurrent infections, and failure to thrive. However, diabetes and skin diseases were not observed in the patient. The patient was received metronidazole, teicoplanin, fluconazole, mycamine, ceftriaxone, azithromycin, and fecal microbiota transplantation for treating infections, methylprednisolone and infliximab for suspicion of Crohn’s disease after admission. Finally, the boy was diagnosed as IPEX syndrome by genetic test and received hematopoietic stem cell transplantation (HSCT). Our findings suggests that IPEX should be considered in cases of late-onset, mild forms, and less typical clinical manifestations to avoid diagnostic delay.

## Introduction

Immune dysregulation, polyendocrinopathy, enteropathy, X-linked (IPEX) syndrome is a rare disorder of genetic autoimmunity, which caused by mutations in the forkhead box protein 3 (FOXP3) gene (1). FOXP3 gene is located in the centromeric region of the X chromosome and encodes a vital regulate factor which required for the function of CD4^+^CD25^+^ regulatory T cells (1). It has been reported that FOXP3 is expressed in tumor cells and involved in the regulation of tumor progression. Studies showed that FOXP3 can act as tumor suppressor in several types of cancer, such as breast cancer (2), prostate cancer (3), and gastric cancer (4). Typical clinical manifestations of IPEX patients are early onset of intractable diarrhea, type 1 diabetes mellitus, as well as skin diseases (1). However, numerous of other autoimmune problems may present that complicate the diagnosis of IPEX, such as severe food allergies, autoimmune cytopenias, autoimmune respiratory disease, and mesangial glomerulonephritis (5). Currently, increasing atypical cases were diagnosed by FOXP3 gene sequencing which indicating the vary expressivity of IPEX syndrome (5). Although IPEX syndrome is often early onset with manifestations appear in the first days of life or even at birth, it has been reported that the onset of the disease could be delayed to early childhood or adolescence (5, 6). Currently, the therapeutic approaches to IPEX syndrome include supportive care and replacement therapy, immunosuppressive treatment, and allogeneic hematopoietic stem cell transplantation (HSCT) (5, 7). However, HSCT is the only curative therapy for IPEX syndrome. HSCT can achieve good outcome when performed at early stage of the disease (5, 8). Thus, early diagnosis is very important for the IPEX syndrome management. Here, we report a 6-year-old boy diagnosed as late-onset IPEX syndrome and treated with HSCT.

## Case Presentation

**Figure 1:**
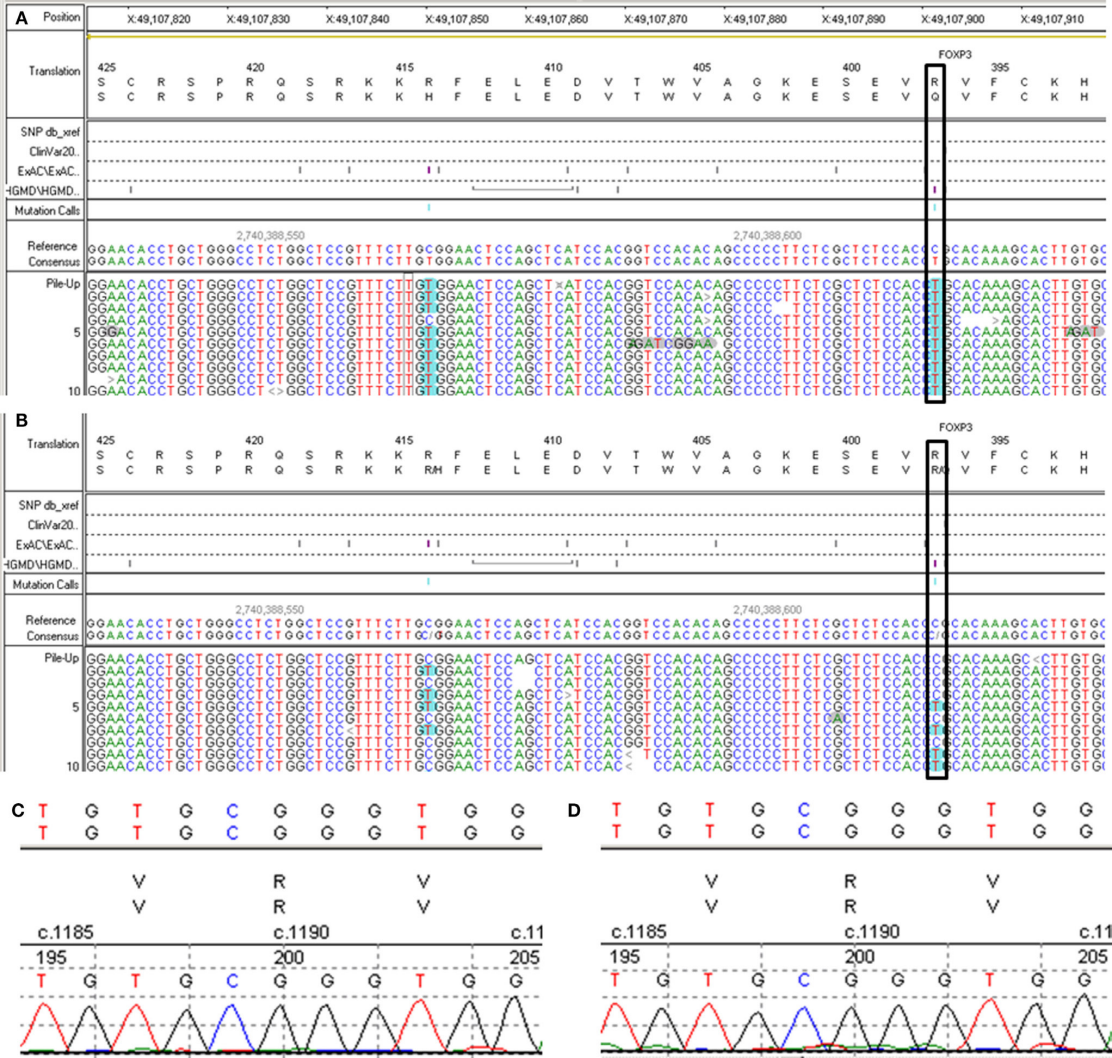
Second generation sequencing for FXOP3 gene mutation identification and DNA Sanger sequencing for the healthy siblings. **(A)** Proband. **(B)** The mother. **(C)** Older brother (Donor). **(D)** Younger sister.

A 6-year- and 9-month-old boy was referred to our hospital with a history of 6-month intractable diarrhea. Diarrhea was characterized by loose, yellow–green, or green, non-bloody, two to three times per day. The boy was born at term, and his family history was normal. Before admission to our hospital, the patient suffered aggravated watery diarrhea for 15 days, 10 times daily, abdominal pain, 5 days of fever, and a weight loss of 3 kg. Laboratory tests showed a normal eosinophil counts, normal immunoglobulin G, IgA, IgM, and IgE levels. Lymphocyte subsets analysis revealed normal proportions of CD3, CD8 T cells, decreased proportions of CD19 T cells (5.74%, reference range: 14.35–22.65%) and CD4 T cells (25.77%, reference range: 29.78–39.94%) (Table 1). A slightly low absolute natural killer cells (0.26 × 10^9^/L, reference range: 0.28–0.63 × 10^9^/L) and a low number of CD4 T cells (0.32 × 10^9^/L, reference range: 0.71–1.84 × 10^9^/L) were observed (Table 1). Antineutrophil cytoplasmic and other autoantibodies, including diabetes-related autoantibodies, antithyroglobulin were all negative (Table 1). The patient was treated with cefoperazone–sulbactam for intestinal infection. Five days later, gastroscopy and colonscopy were performed for no improvement of symptoms. Colonscopy showed mucosal damages and ulceration, biopsy histology showed eosinophil infiltration (20–30/HP), and hyperplasia of lymphoid follicles. The patient was started with methylprednisolone for suspicion of Crohn’s disease based on colonic ulcers and biopsy pathological features. Infliximab (5 mg/kg) was given for no significant remission after methylprednisolone treatment. Then, the patient was discharged for achieving a partial remission of diarrhea. Two weeks later, he was admitted to our hospital again for second course of infliximab. Diarrhea was still presented in the patient, and characterized by bloody, mucus-containing stools. Metronidazole was started due to *Clostridium difficile* infection (CDI). Oral methylprednisolone and deep hydrolyzed milk were given and discharged for partial improvement of symptom. Two weeks before the third admission, the boy suffered with severe watery diarrhea again, with occasionally bloody, mucus containing stools, more than 10 times per day, accompanied with abdominal pain and fever. During the third admission, vancomycin and teicoplanin were used to treat CDI, fluconazole and mycamine were used to treat *Candida albicans* infection which found in the mouth, ceftriaxone, and azithromycin were given to treat pneumoniae *Mycoplasma*. In addition, fecal microbiota transplantation (FMT) was performed to treat CDI (9). Albumin, gamma globulin, red cell suspension, and frozen plasma were given due to hypoproteinemia. Parenteral nutrition and enteral nutrition were supported for failure to thrive. Diarrhea, abdominal pain, mucus-containing stools, and recurrent infections were not well controlled after more than 2 months of continuous treatment. Therefore, the patient was suspicious of immunodeficiency disease and extraction of peripheral blood for immune gene panel testing. Finally, the boy was diagnosed as IPEX syndrome due to a c.1190G>A (p. R397Q) mutation in exon 11 of the FOXP3 gene (Figure 1A). The patient’s mother is a healthy carrier (Figure 1B). Two healthy siblings were excluded the same mutation by DNA Sanger sequencing (Figures 1C,D). At 3 months after the genetic diagnosis of IPEX, at the age of 7.5 years, the boy underwent matched sibling peripheral blood HSCT using reduced-intensity conditioning with busulfan, fludarabine, and cyclophosphamide, antithymocyte globulin. The CD34 cell dose was 7 × 10^6^/kg, and the number of neutrophils was 3.09 × 10^9^/L, platelets was 276 × 10^9^/L in the day of engraftment. He continued to receive cyclosporine and methylprednisolone to prevent graft-versus-host disease. At day +14 post-HSCT, the short tandem repeat test showed that he engrafted with 100% of donor cells. Up to date, diarrhea, abdominal pain, and recurrent infections were well controlled after 6 months of HSCT, and *Clostridium difficile* (CD) test was negative.

**Table 1:** Blood count, lymphocyte subsets, and tested antibodies.

Blood index	Count (×10^9^)	Ratio (%)
Red blood cell (RBC)	4.25	
white blood cell (WBC)	12.52	
Hemoglobin (Hb; g/L)	104	
Platelet (PLT)	586	
Neutrophil (N)		82
Lymphocyte (L)		11
CD3	0.90	72.44
CD4	0.32	25.77
CD8	0.49	39.98
CD16CD56CD3^−^	0.26	21.21
CD19	0.07	5.74
**Autoantibodies**	**Result**	
Anti-dsRNA antibody	−	
Anti-ribonucleoprotein antibody	−	
Anti-SM antibody	−	
Anti-sicca syndrome A antibody	−	
Anti-sicca syndrome B antibody	−	
Anti-ScL-70 antibody	−	
Anti-centromere protein B antibody	−	
Anti-Jo-1 antibody	−	
Anti-proliferation cell nuclear antigen antibody	−	
Anti-perinuclear antineutrophil cytoplasmic antibody	−	
Anti-antineutrophilic cytoplasmic antibody	−	
Anti-mitochondrial antibody	−	
Anti-histone antibody	−	
Anti-ribosomal P protein antibody	−	
Anti-PM-ScL antibody	−	
Anti-nucleosome antibody	−	
Antithyroglobulin antibody	−	
Anti-insulin antibodies	−	
Anti-insulin cell antibodies	−	

*ScL, sclerosis; PM, polymyositis; −, negative*.

## Discussion

Although IPEX syndrome is characterized by early-onset, typical clinical manifestations of enteropathy, type 1 diabetes mellitus, and skin diseases, the number of diagnosed atypical IPEX cases was increased in recent years (5). Late-onset, mild forms, or less common clinical manifestations are easily causing delayed diagnosis in atypical IPEX patients (5). In this report, we presented a late-onset IPEX syndrome case with chronic diarrhea as first symptom in the age of 6 years. The patient had a long history of intractable diarrhea, and suffered infections with CD, *Candida albicans*, and *Mycoplasma*. The patient was received metronidazole, vancomycin, teicoplanin, fluconazole, mycamine, ceftriaxone, azithromycin, and FMT for treating infections, methylprednisolone and infliximab for suspicion of Crohn’s disease at three times of admission in 2.5 months. However, diarrhea, abdominal pain, mucus-containing stools, intestinal inflammation, and recurrent infections were not controlled. Finally, the patient was considered as immunodeficiency disease and diagnosed as IPEX syndrome. A c.1190G>A (p. R397Q) mutation in the FOXP3 was observed by immune gene panel testing. This finding suggested the importance of consideration of IPEX syndrome in older child with less typical common clinical manifestations to avoid delayed diagnosis.

Currently, numerous studies have reported more than 70 distinct FOXP3 mutations in IPEX syndrome patients (5). However, there is no clear genotype–phenotype correlation. Identical FOXP3 mutations can cause significantly different phenotypes in different patients. Our case was the third reported IPEX syndrome caused by c.1190G>A (p. R397Q) mutation. Different clinical manifestations were presented in these three IPEX cases. Tsuda et al. reported an IPEX case of 4-year-old boy caused by c.1190G>A (p. R397Q) mutation had most of the common clinical symptom of enteropathy, skin disease and type 1 diabetes mellitus (10). An early-onset IPEX case of 1-month-old boy was reported due to c.1190G>A (p. R397Q) mutation with a history of watery diarrhea, severe hyponatremic dehydration, and dermatitis (11). While the case we presented here was a late-onset IPEX syndrome with manifestation of intractable diarrhea and recurrent infections with c.1190G>A (p. R397Q) mutation in FOXP3 gene.

Currently, there is no standardized therapeutic approach to IPEX syndrome due to limited reported cases and lacking of multicentric studies (5). Immunosuppressive treatment and allogeneic HSCT were mostly used to treat IPEX syndrome. So far, HSCT is the sole curative therapy option for IPEX syndrome with a high survival rate and can archive the best outcome when performing at early stage of the disease (8). It was reported that more than 30 IPEX patients received HSCT (5). In this report, the patient underwent matched sibling peripheral blood HSCT at age of 7.5 years after 3 months of the genetic diagnosis of IPEX. Up to date, the enteropathy had been completely cured, and all clinical manifestations were in remission after 6 months of HSCT. The long-term effect of HSCT on the clinical outcome of the patient needs further evaluation.

In summary, our finding suggests that IPEX should be considered in the cases of late-onset, mild forms, and less typical clinical manifestations to avoid diagnostic delay.

## Ethics Statement

Written informed consent for the presentation and publication of this case was obtained from the patient’s parents.

## Author Contributions

TG and YW drafted the manuscript. RC, YM, and TZ acquired, analyzed, and interpreted the data. TZ edited the manuscript.

## Conflict of Interest Statement

The authors declare that the research was conducted in the absence of any commercial or financial relationships that could be construed as a potential conflict of interest.
